# Intraoperative Assessment of the Resection Specimen Facilitates Achievement of Adequate Margins in Oral Carcinoma

**DOI:** 10.3389/fonc.2020.614593

**Published:** 2020-12-23

**Authors:** Roeland W. H. Smits, Cornelia G. F. van Lanschot, Yassine Aaboubout, Maria de Ridder, Vincent Noordhoek Hegt, Elisa M. Barroso, Cees A. Meeuwis, Aniel Sewnaik, Jose A. Hardillo, Dominiek Monserez, Stijn Keereweer, Hetty Mast, Ivo Ten Hove, Tom C. Bakker Schut, Robert J. Baatenburg de Jong, Gerwin J. Puppels, Senada Koljenović

**Affiliations:** ^1^ Department of Otorhinolaryngology and Head and Neck Surgery, Erasmus MC Cancer Institute, University Medical Center Rotterdam, Rotterdam, Netherlands; ^2^ Center for Optical Diagnostics and Therapy, Department of Dermatology, Erasmus MC Cancer Institute, University Medical Center Rotterdam, Rotterdam, Netherlands; ^3^ Department of Pathology, Erasmus MC Cancer Institute, University Medical Center Rotterdam, Rotterdam, Netherlands; ^4^ Department of Medical Informatics, Erasmus MC Cancer Institute, University Medical Center Rotterdam, Rotterdam, Netherlands; ^5^ Department of Oral and Maxillofacial surgery, Special Dental Care, and Orthodontics, Erasmus MC Cancer Institute, University Medical Center Rotterdam, Rotterdam, Netherlands

**Keywords:** cancer, resection margins, intraoperative assessment, specimen-driven, follow-up

## Abstract

**Background:**

Inadequate resection margins in oral cavity squamous cell carcinoma have an adverse effect on patient outcome. Intraoperative assessment provides immediate feedback enabling the surgeon to achieve adequate resection margins. The goal of this study was to evaluate the value of specimen-driven intraoperative assessment by comparing the margin status in the period before and the period after the introduction of specimen-driven assessment as a standard of care (period 2010–2012 vs period 2013–2017).

**Methods:**

A cohort of patients surgically treated for oral squamous cell carcinoma at the Erasmus MC Cancer Institute, Rotterdam, between 2010–2012 was studied retrospectively and compared to results of a prospectively collected cohort between 2013–2017. The frequency, type and results of intraoperative assessment of resection margins were analyzed.

**Results:**

One hundred seventy-four patients were included from 2010–2012, 241 patients were included from 2013–2017. An increase in the frequency of specimen-driven assessment was seen between the two periods, from 5% in 2010–2012 to 34% in 2013–2017. When performing specimen-driven assessment, 16% tumor-positive resection margins were found in 2013–2017, compared to 43% tumor-positive resection margins overall in 2010–2012. We found a significant reduction of inadequate resection margins for specimen-driven intraoperative assessment (p < 0.001). Also, tumor recurrence significantly decreased, and disease-specific survival improved when performing specimen-driven intraoperative assessment.

**Conclusions:**

Specimen-driven intraoperative assessment improves resection margins and consequently, the outcome of oral cancer patients. We advocate this method as standard of care.

## Introduction

Patients with inadequate tumor resection margins often receive adjuvant treatment (radiotherapy, chemoradiation and/or re-operation), which leads to higher morbidity (1).

Moreover, inadequate resection margins in oral cavity squamous cell carcinoma (OCSCC) lead to a significantly worse clinical outcome (2–4).

In our previous retrospective study, we found inadequate resection margins (i.e., a distance of ≤5 mm from tumor border to resection surface) in 85% of OCSCC cases based on final histopathology (3). Equally low numbers of adequate OCSCC resections were reported by other authors (2, 4).

This illustrates that for the oral cavity, with its complex anatomy, inspection and palpation by the surgeon during the operation are often insufficient to warrant an adequate resection.

In order to control resection margins, intraoperative assessment by frozen sectionprocedure is available. During this procedure, the surgeon samples tissue from seemingly the most suspicious areas in the wound bed (i.e., the defect-driven intraoperative assessment). For the detection of inadequate margins during OCSCC surgery, this defect-driven frozen section procedure has been shown to have low sensitivity (5–9). Moreover, this procedure is time-consuming and only a limited number of tissue samples can be examined, leading to sampling error, and resulting in underestimation of inadequate margins (10–15). Furthermore, the defect-driven frozen section procedure cannot provide the exact length of resection margins (in millimeters); it can only indicate the presence of tumor-positive margins.

To overcome these limitations, the specimen-driven intraoperative assessment, performed by the surgeon and pathologist together, has been advocated. This approach provides immediate feedback on whether an additional resection is needed. Recent studies show that this type of intraoperative assessment is superior to defect-driven assessment due to better visualization, less sampling error and it has been recommended in the latest AJCC guidelines (4, 6, 16–21).

At our institute, this multidisciplinary approach has been introduced in 2013.

This study aimed to evaluate the value of specimen-driven intraoperative assessment by comparing the margin status in the period before and the period after the introduction of specimen-driven assessment (i.e., period 2010–2012 vs period 2013–2017).

## Material and Methods

### Patient Selection

The study was approved by the institutional Medical Ethics Committee (MEC-2015-150). All patients treated surgically for OCSCC in the period from October 2010–October 2012 and September 2013–January 2017 were selected for analysis.

The period from 2010–2012, when specimen-driven intraoperative assessment was not standard of care, has been described earlier (3).

### Data Collection

A database was created containing patient characteristics (i.e., age, gender, comorbidity, smoking habit), and tumor characteristics (i.e., subsite, pathological TNM classification, differentiation grade, perineural growth, pattern of invasion).

In addition, margin status was recorded, based on both; intraoperative assessment and final histopathology. The type of intraoperative assessment was recorded as defect-driven or specimen-driven. The margins were defined based on the guidelines of the Royal College of Pathologists: >5 mm as clear, 1-5 mm as close and <1 mm as tumor-positive (22). Clear margins are referred to as adequate, close and tumor-positive margins as inadequate. All cases were reviewed by one or two dedicated head and neck pathologists (S.K., V.N.H.).

Follow up data was collected from the patient files until 27-09-2019. Data on local recurrence, regional recurrence and distant metastasis were recorded. Mortality was also recorded, including the cause of death to calculate disease-specific survival (DSS).

### Specimen-Driven Intraoperative Assessment

**Figure 1:**
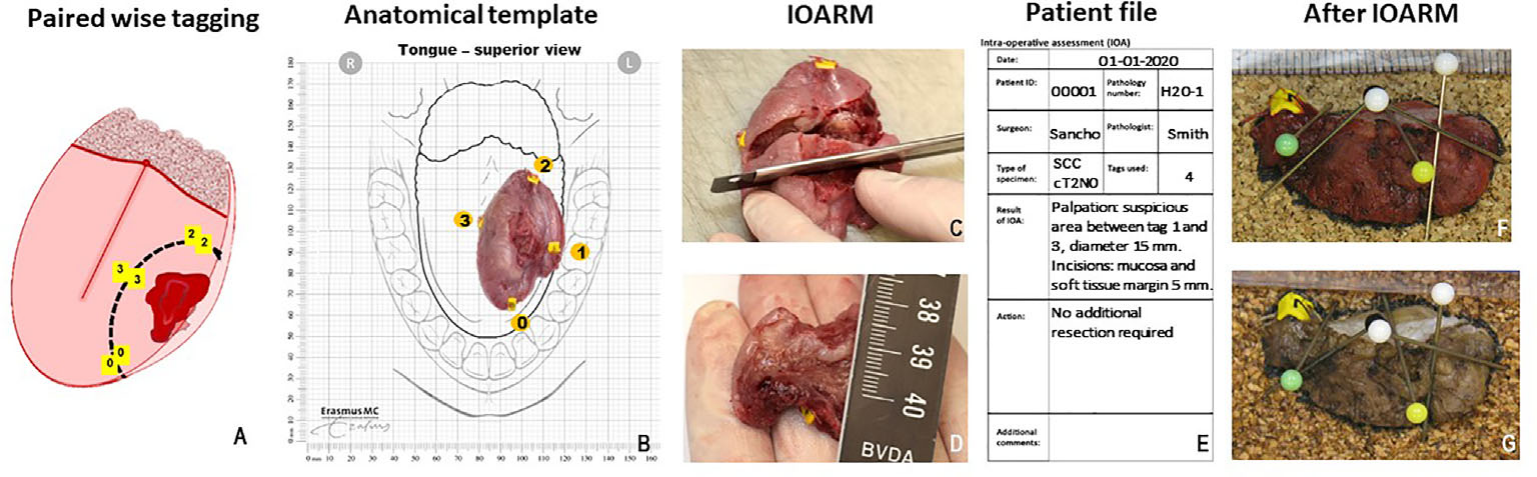
**(A)** Paired wise tagging on both sides of the resection line, performed during surgery (23). **(B)** Anatomical template, used to maintain orientation, tags are noted on the template. **(C)** Grossing of the tissue, perpendicular incisions must be 5–6 mm from each other. **(D)** Measuring the margin with a ruler. **(E)** Patient file, used for patient information, reporting results and recommendations. **(F)** Cross section of fresh tissue placed against cork to maintain shape and orientation during fixation. **(G)** Cross section after fixation shows no shrinkage of tissue or change in shape.

**Figure 1** shows an example of the specimen-driven IOARM procedure. During operation, the surgeon places numbered tags in a pair-wise manner on both sides of the resection line, both superficially and deep in the wound bed (**Figure 1A**). When the resection is completed, one tag of each pair remains attached to the specimen and the other tag stays in the wound bed. These tags are later used to relocate an inadequate margin in the wound bed. This relocation method was described in more detail by van Lanschot et al. (23).

Next, the specimen is taken to the pathology department for intraoperative assessment. The surgeon and the pathologist select an anatomical template that best illustrates the anatomical orientation of the resection specimen and wound bed (**Figure 1B**). The pathologist and surgeon visually inspect and palpate the specimen to locate suspicious areas (i.e., areas on the resection surface that might have an inadequate margin). If a suspicious area is found, the pathologist makes one or more parallel (partial or complete) incisions, perpendicular to the tissue surface with a mutual distance of approximately 5 mm (**Figure 1C**).

In most cases, this enables the visualization and measurement of the margin of healthy tissue on the cross-sectional side with a ruler (**Figure 1D**).

If no inadequate margins are found, the surgeon can return to the operating room and close the wound. If an inadequate margin is detected on the specimen, the numbered tags enclosing such area are used by the surgeon to detect this area in the wound bed. It can then be determined if an additional resection is possible. The required thickness of the additional resection is indicated by the pathologist (in millimeters). For example, if the initial margin is 2 mm, the pathologist recommends an additional resection of tissue with at least 4 mm thickness to achieve a margin of more than 5 mm.

The whole specimen-driven IOARM process, including the conclusion and the recommendation for additional resection, is recorded and stored in the patient file (**Figure 1E**).

Next, to maintain the anatomical orientation and shape of the specimen, tissue cross sections created for intraoperative assessment are placed between two pieces of cork at the original location in the specimen, and held in place by needles (**Figures 1F, G**) prior to formalin fixation.

After the intraoperative assessment, the resection specimen enters the routine procedure for the final pathological examination.

### Statistical Analysis

Differences in patient and tumor characteristics between the two periods (2010–2012 vs 2013–2017) were tested with t-test for continuous variables and with a chi-square test for categorical variables. Differences between the three intraoperative assessment types (i.e., “no intraoperative assessment”, “defect-driven assessment”, and “specimen-driven assessment”) were tested with a one-way ANOVA for continuous variables and with a chi-square test for categorical variables.

Differences in achieving adequate resection margins comparing IOARM groups were estimated with Poisson regression with robust standard errors. Crude relative risks (RR) for defect-driven assessment and specimen-driven assessment compared to no intraoperative assessment were estimated as well as RRs adjusted for gender, age, tumor size and location. Tumor subsites were: tongue, floor of mouth, alveolar process, retromolar trigone and palate. Because of the low number of patients with tumors located at the retromolar trigone and palate we decided to merge these two groups into the group ‘other’ for statistical analysis.

Time to local recurrence within three years after surgery was described with Kaplan-Meier estimations, and compared between groups based on margin status (i.e., >5 mm “clear”, 1–5 mm “close” and <1 mm “tumor-positive”) with a logrank test for trend. For comparing time to all recurrence events (local recurrence, regional recurrence, distant metastasis) complete follow-up was analysed. For disease-specific survival, events within 2 months after surgery were omitted to exclude surgery-related mortality.

## Results

### 2010–2012

During this period, 174 patients were treated surgically for OCSCC at the Erasmus MC Cancer Institute. Patients and tumor characteristics are shown in **Table 1**.

**Table 1:** Patient characteristics.

	2010–2012 n = 174	2013–2017 n = 241	p-value difference
**Median age (range)**	65 (16–93)	67 (24–95)	0.09
**Male, %**	68	53	0.002
**pT1-pT2, %**	53	71	<0.001
**Subsite, %**			0.03*
**-tongue** **-floor of mouth** **-alveolar process** **-cheek** **- lip** **- other**	41 27 27 5 0 0	46 22 17 8 1 6	

*Difference tested after re-categorization to “tongue,” “floor of mouth,” “mandible,” and “other.”

IOARM was performed during 24 operations (14%), with defect-driven assessment in 16 cases (9%) and specimen-driven in 8 cases (5%) (**Table 2**).

**Table 2:** Frequency and type of intraoperative assessment of resection margins.

Type of intraoperative assessment of resection margins	2010–2012 (n = 174)	2013–2017 (n = 241)
Defect-driven	9%	27%
Specimen-driven	5%	34%
Total	14%	61%

Upon final histopathological evaluation, adequate resection margins were found in 15% of cases, close resection margins in 42%, and tumor-positive resection margins in 43% of cases. Resection margins status per subsite are shown in **Table 3**.

**Table 3:** Resection margin status per subsite based on final pathology.

	Adequate		Close		Tumor-positive	
	2010–2012	2013–2017	2010–2012	2013–2017	2010–2012	2013–2017
	n = 26	n = 78	n = 73	n = 101	n = 75	n = 62
**-tongue** **-floor of mouth** **-alveolar process** **-cheek** **-lip** **-other**	15 (21%) 8 (16%) 1 (5%) 1 (8%) 0 (0%) 1 (5%)	51 (46%) 11 (21%) 9 (22%) 3 (15%) 3 (100%) 1 (7%)	40 (56%) 18 (36%) 7 (37%) 3 (25%) 0 (0%) 5 (24%)	47 (42%) 24 (45%) 14 (34%) 10 (53%) 0 (0%) 6 (43%)	17 (23%) 24 (48%) 11 (58%) 8 (67%) 0 (0%) 15 (71%)	13 (12%) 18 (34%) 18 (44%) 6 (32%) 0 (0%) 7 (50%)

### 2013–2017

In this period, 241 patients were treated surgically for OCSCC at the Erasmus MC Cancer Institute. Patients and tumor characteristics are shown in **Table 1**.

IOARM was performed in 146 cases (61%), as shown in **Table 2**.

Defect-driven intraoperative assessment was performed in 65 cases (27%), specimen-driven in 81 cases (34%).

Upon final histopathological evaluation, adequate resection margins were found in 32% of cases, close resection margins in 42%, and tumor-positive resection margins in 26% of cases. Resection margins status per subsite are shown in **Table 3**.

All cases, for both periods were subdivided into three IOARM groups; 1) no intraoperative assessment, 2) defect-driven assessment, and 3) specimen-driven assessment. The results are shown in **Table 4**.

**Table 4:** Resection margin status in relation to IOA based on final pathology.

	None		Defect-driven		Specimen-driven	
	2010–2012	2013–2017	2010–2012	2013–2017	2010–2012	2013–2017
	n = 150	n = 95	n = 16	n = 65	n = 8	n = 81
**-adequate** **-close** **-tumor-positive**	24 (16%) 62 (41%) 64 (43%)	16 (17%) 49 (52%) 30 (31%)	2 (12.5%) 6 (37.5%) 8 (50%)	15 (23%) 31 (48%) 19 (29%)	0 (0%) 3 (37.5%) 5 (62.5%)	47 (58%) 21 (26%) 13 (16%)

### Impact of Intraoperative Assessment

The impact of intraoperative assessment was investigated only from September 2013, when the comprehensive specimen-driven IOARM protocol was implemented.

Patient characteristics did not differ between the IOARM groups. When comparing tumor characteristics, significant differences were found for the subsite of the tumor, with the specimen-driven assessment group having more tumors located at the tongue, and fewer tumors located at the alveolar process and at the ‘other’ subsite (P = 0.05).

The crude relative risk of inadequate resection margins for defect-driven assessment compared to no intraoperative assessment was not significant (RR 0.93, 95% CI 0.79 to 1.09). Comparison between specimen-driven assessment and no intraoperative assessment was significant (RR 0.51, 95% CI 0.39 to 0.66). Adjusted RR of inadequate margins for defect-driven assessment was 0.93 (95% CI 0.79 to 1.09) and for specimen-driven 0.54 (95% CI 0.41 to 0.71). The results are listed in **Table 5**.

**Table 5:** Effect of intraoperative assessment on inadequate resection margins.

		Unadjusted model			Adjusted model*		
		RR	95% CI	p-value	RR	95% CI	p-value
**IOARM**	None	ref		< 0.001	ref		< 0.001
	Defect-driven	0.93	0.79, 1.09		0.93	0.79, 1.09	
	Specimen-driven	0.51	0.39, 0.66		0.54	0.41, 0.71	

*Adjusted for gender, age, tumor size, and location.

### Specimen-Driven Intraoperative Assessment

The accuracy of specimen-driven IOARM was calculated by comparison of margin status based on IOARM and that from final histopathology. This resulted in an overall accuracy of 63.1%.

**Figure 2:**
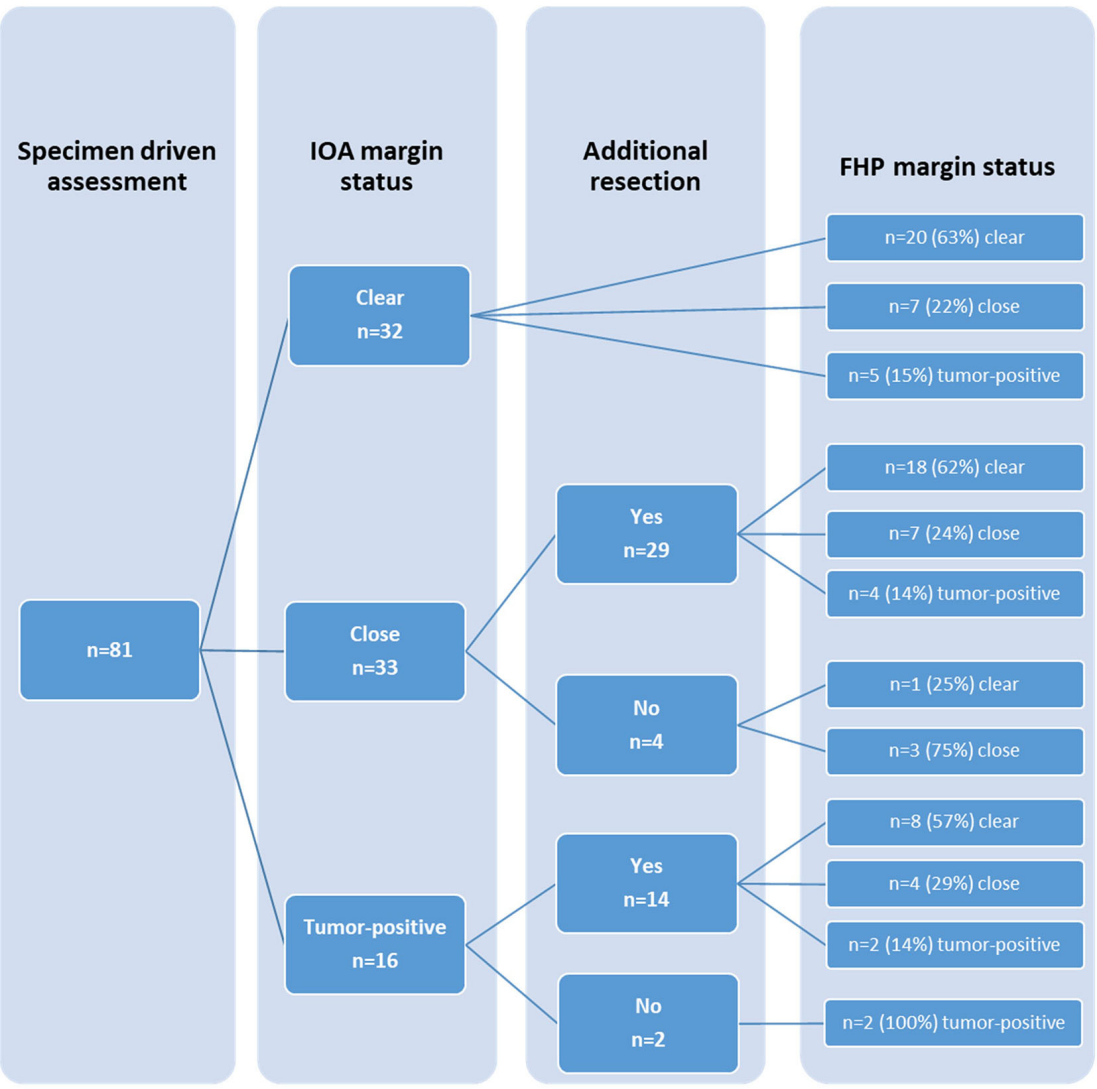
Comparison of margin status based on intraoperative assessment (IOA) and margin status based on final histopathology (FHP), including additional resection.

Final margin status, with or without additional resection, is shown in **Figure 2**.

In 43 cases an additional resection was performed based on specimen-driven IOARM. In 30 cases additional resection resulted in improvement; 26 from close to clear margin, and 4 cases from positive to close margin. In the remaining 13 cases margins did not improve after additional resection.

In six cases inadequate margins were identified during IOARM but additional resection was not performed because of close proximity of vital structures.

### Tumor Recurrence Rate and Survival Based on Margin Status

**Figure 3:**
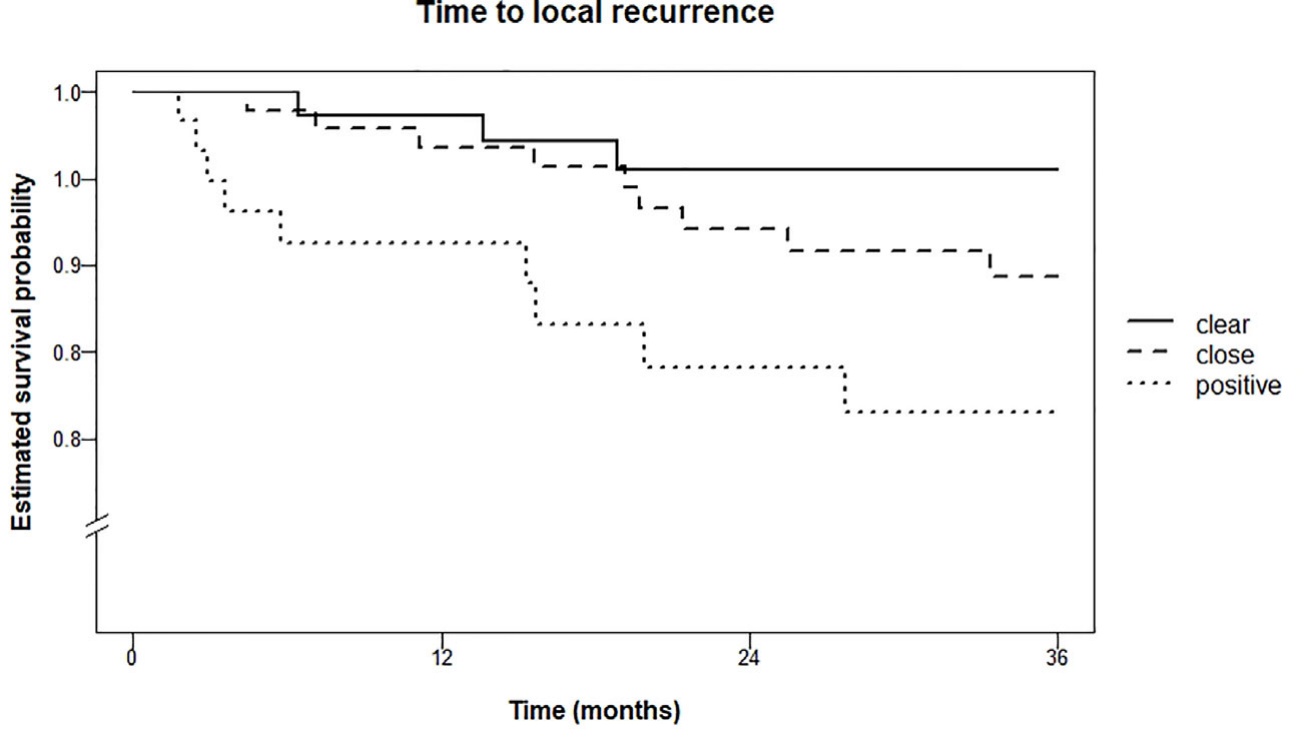
Kaplan Meier estimations of time to local recurrence in months.

Local recurrence rate within three years was 4.5% for patients with clear resection margins, 10.6% in the group with close resection margins, and 18.5% in the group with tumor-positive resection margins (logrank test for trend P = 0.01). Kaplan Meier curves are shown in **Figure 3**.

**Figure 4:**
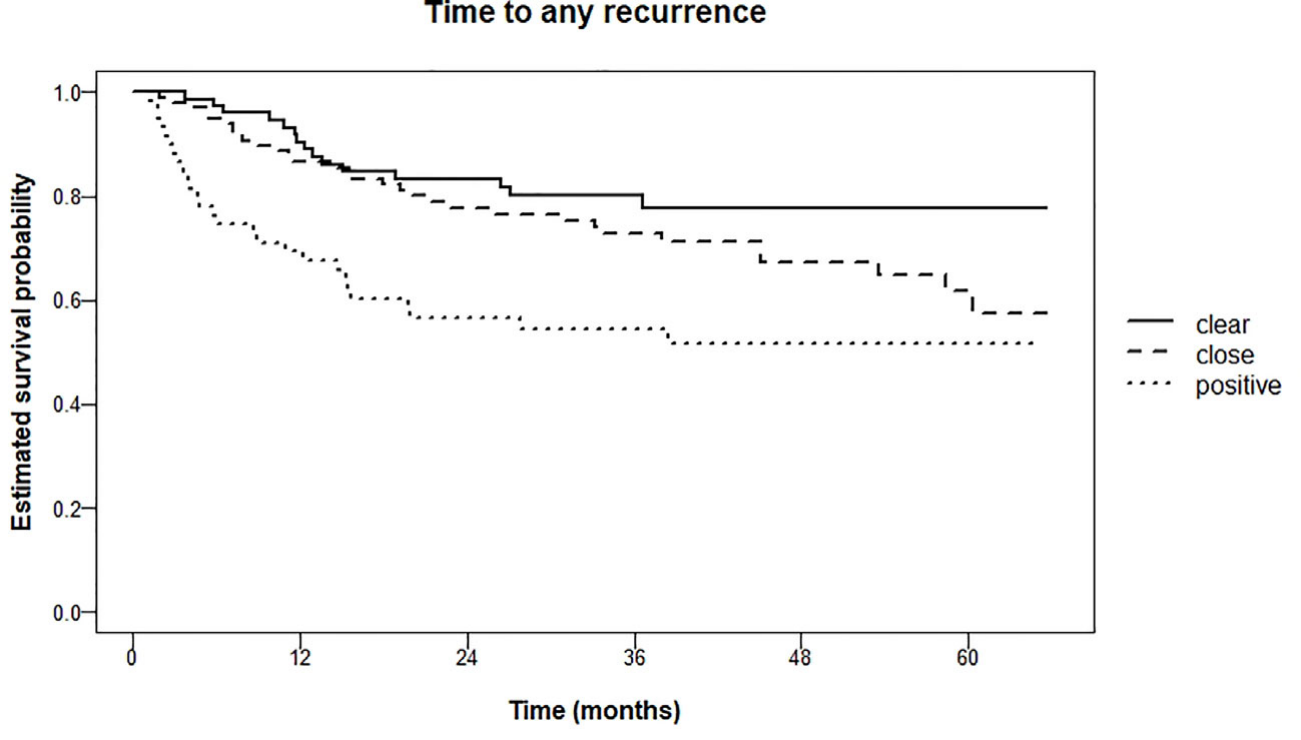
Kaplan Meier estimations of time to any recurrence (local, regional, distant metastasis) in months.

The difference in occurrence of any recurrence (i.e., local, regional, distant) within 5 years was significant (logrank test for trend P = 0.001) between the three groups; 22.2% (clear), 38.3% (close) and 48.2% (tumor-positive). Kaplan Meier curves are shown in **Figure 4**.

**Figure 5:**
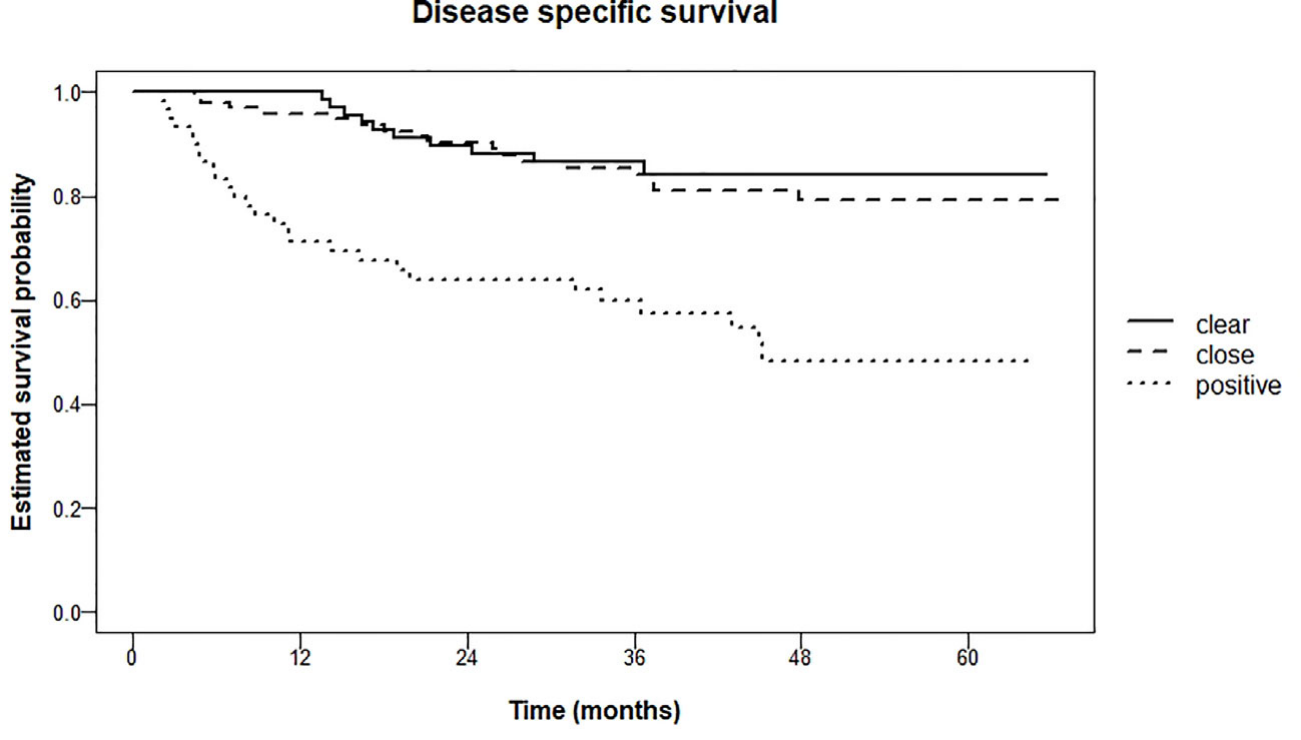
Kaplan Meier estimations of disease-specific survival in months.

For disease-specific survival these percentages after 5 years were 15.7% (clear), 20.9% (close) and 51.7% (tumor-positive) respectively (logrank test for trend P <0.001). Pairwise comparison of clear resection margins and close resection margins showed no significant difference (P = 0.60). However, when comparing clear resection margins with tumor-positive resection margins, and close resection margins with tumor-positive resection margins, there was a significant difference (both P < 0.001). Kaplan Meier curves are shown in **Figure 5**.

## Discussion

Of all the prognostic factors (i.e., patient and tumor characteristics) in oncological patients, surgeons and pathologists can only influence the resection margins. Adequate resection of OCSCC, as for many other tumors, is sometimes hard to achieve because of a lack of reliable intraoperative guidance and the complex anatomy of the oral cavity. These are some of the explanations why multiple studies showed a high number of inadequate resection margins for OCSCC (2, 4).

To improve the status of resection margin at our institute, a comprehensive specimen-driven intraoperative assessment of resection margins has been implemented in September 2013. The procedure is performed by a dedicated team of head and neck surgeons and pathologists.

The frequency of intraoperative assessment increased from 14% for the period before 2013 compared to 61% in the period after 2013, irrespective of the assessment type. Moreover, since 2013, for OCSCC, specimen-driven intraoperative assessment was performed almost seven times more often compared to the period before 2013 (34 vs. 5%). Furthermore, we saw an increase of specimen-driven intraoperative assessment from 12% in 2013 to 54% in 2017.

Comparing the resection margin status of all cases from both periods (2010–2012 and 2013–2017), with or without intraoperative assessment, we found an increase of adequate margins from 15 to 32% and a decrease in tumor-positive resection margins from 43 to 26%. Further improvement was achieved when specimen-driven intraoperative assessment was performed: 58% adequate margins and only 16% tumor-positive margins were found after 2013. A decrease of tumor-positive margins was also seen when defect-driven intraoperative assessment was performed: from 50 to 29%. This can be explained by a increase of awareness of the head and neck surgeons who participated in this study. Since our retrospective study where we showed 85% inadequate margins overall, the head and neck surgeons confirmed that they started to be more aware of inadequate margins (3). This can explain the fact that tumor-positive resection margins decreased in all groups, even in the group without intraoperative assessment. The decrease of the number of tumor-positive margins was highest in the specimen-driven assessment group (62.5 to 16%.

The inadequate margins found when analyzing specimen-driven intraoperative assessment from 2010–2012 are partly caused by the fact that we only started performing an extensive specimen-driven approach (as illustrated in this paper) in 2013. In the period 2010-2012 specimen-driven method was not optimal, and was only performed in eight cases, compared to 81 cases from 2013–2017.

As we have shown, adequate margins result in lower rates of local recurrence, regional recurrence, and distant metastasis. Also, disease-specific survival is significantly higher for patients with adequate margins.

This is in accordance with other studies (4, 6, 18–20).

We therefore advocate specimen-driven assessment as standard of care during OCSCC surgery. This is in line with the latest guidelines of the AJCC (16).

There is a number of possible sources of bias in this study. During surgery, it can become evident that achieving adequate resection margins is virtually impossible due to close proximity of vital structures.Although peroperative planning is of essential importance, it unfortunately does not always reflect the intraoperative situation. Preoperative images are often made weeks prior to surgery and tumor may expand in the meantime. Because complete tumor resection (R0) remains the aim of surgery, most structures in the oral cavity can be sacrificed to obtain adequate margins.On contrary, doubt about tumor invasion in of for instance major head and neck nerves or the mandible, can pose surgeon to a difficult choice at that moment, when adequate margins are warranted.

Therefore, achieving adequate resection margins can be more difficult for some locations within the oral cavity. For tongue and lip it seems to be easier to achieve an adequate margin than, for instance, for hard palate or floor of mouth, as shown in **Table 3**. As there were significantly more tumors of the tongue in the specimen-driven assessment group, this could influence the results. Therefore, we have adjusted results for patient and tumor characteristics, including tumor subsite.

There are limitations of specimen-driven IOARM that need to be addressed. Grossing fresh tissue is counter-intuitive to pathologists because it is more difficult than grossing fixated tissue. Grossing fresh tissue might affect the anatomical orientation and shape of the specimen, which in turn might affect final pathology assessment (24, 25). Our specimen-driven IOARM protocol addresses this by digitally recording every step of the procedure, including the grossing of the specimen and its reconstruction on cork plates, for preservation of anatomical orientation and shape. We have not observed changes in shape or size (shrinkage) of cross sections after fixation, and we have not encountered a single case in which final pathology was affected in any way.

Performing the specimen-driven IOARM, as described here, takes additional time. We estimate that, on average, 30 min is needed including transfer of the specimen to the pathology department. In this time, sometimes the surgical procedure can be continued by performing a neck dissection, but in other cases the procedure has to be put on hold until results of IOARM are known.

Perhaps the most critical limitation of IOARM is that the method remains subjective and only a limited number of incisions can be placed on freshly resected specimen so as not to interfere with final histopathological evaluation. We found 63.1% overall accuracy of IOARM, which means that there is room for improvement.

A potential limitation of the current study is the fact that for close resection margins we use the definition of the Royal College of Pathologists, 1–5 mm. In recent years there has been much debate about the optimal resection margin for OCSCC (26). Several authors suggest that resection margins between 2–3 mm could be sufficient while not hampering patient outcome (27–29). Still, no change of guidelines has been made, so for this study, we have chosen to stay with the 1–5 mm definition.

There is a learning curve to go through. For the pathologist, this learning curve comprises discriminating salivary gland tissue and scar tissue from tumor upon palpation and inspection, and to refine the procedure by microscopic evaluation of frozen sections. Another important aspect of the learning process is the meticulous handling of the tissue before fixation. However, the most important prerequisite is close coordination of logistics between surgeons and pathologists. Unfortunately, this will not be feasible for all clinical settings, so alternative methods or techniques should be investigated.

Based on the favourable results presented in this study, and despite its limitations and the additional effort, we strongly advocate the implementation of specimen-driven IOARM in OCSCC surgery.

At the Erasmus MC Cancer Institute, we are currently developing a method for OCSCC surgery guidance based on two optical techniques, fluorescence-guided surgery and Raman spectroscopy (30, 31). The combination of these techniques is being developed to allow for a rapid and accurate specimen-driven intraoperative assessment of all resection surfaces that will fit in the surgico-pathological workflow.

Only by intraoperative assessment of all resection margins, it will be possible to consistently obtain a high number of adequate margins and thereby improve the clinical outcome of OCSCC patients.

## Data Availability Statement

The raw data supporting the conclusions of this article will be made available by the authors, without undue reservation.

## Ethics Statement

The studies involving human participants were reviewed and approved by Erasmus MC Medical Ethics Committee MEC-2015-150. Written informed consent for participation was not required for this study in accordance with the national legislation and the institutional requirements.

## Author Contributions 

RS designed the study, performed intraoperative assessment of resection margins, carried out the retrospective database study, and drafted the manuscript. CL and YA performed intraoperative assessment of resection margins and drafted the manuscript. EB, IH, HM, AS, CM, DM, JH, and SKe performed intraoperative assessment of resection margins and revised the manuscript critically for important intellectual content. RJ participated in the design of the study, revised the manuscript critically for important intellectual content, and gave final approval of the version to be published. TS participated in the design of the study and revised the manuscript critically for important intellectual content. VH was responsible for the histopathological evaluation of the patient material used in this study and drafted the manuscript. GP designed the study, supervised the research group, and revised the manuscript critically for important intellectual content and gave final approval of the version to be published. SKo designed and supervised this study, was mainly responsible for the intraoperative assessment of resection margins, and for the final histopathological evaluation of the patient material used in this study and gave final approval of the version to be published. MR was responsible for the statistical analysis of the data. All authors contributed to the article and approved the submitted version.

## Funding

The authors declare that this study received funding from Atos Medical BV. The funder was not involved in the study design, collection, analysis, interpretation of data, the writing of this article or the decision to submit it for publication.

## Conflict of Interest

The authors declare that the research was conducted in the absence of any commercial or financial relationships that could be construed as a potential conflict of interest.

## Abbreviations

OCSCC, oral cavity squamous cell carcinoma; IOARM, intraoperative assessment of resection margins; FHP, final histopathology; MC, Medical Center; MR, magnetic resonance; CT, computed tomography; ANOVA, analysis of variance; RR, relative risk; CI, confidence interval; TNM, Tumor, lymph nodes, metastasis (according to the TNM Classification of Malignant Tumors); GEE, generalized estimating equation; AJCC, American Joint Committee on Cancer.
